# Case Report: A Case of Post-Transplant Chagas Reactivation after Negative *Trypanosoma cruzi* Testing

**DOI:** 10.4269/ajtmh.22-0313

**Published:** 2023-03-13

**Authors:** Omar Shakhtour, Tsion Aberra, Huzaifa Ahmad, Abhinav Saxena, Imad Isaac, Namratha Meda, Ritika Gadodia, Rachel Marcus

**Affiliations:** 1 Department of Internal Medicine, Medstar Washington Hospital Center, Washington, District of Columbia;; 2 Department of Cardiology, Medstar Washington Hospital Center, Washington, District of Columbia

## Abstract

Patients with Chagas cardiomyopathy carry a significant risk of reactivation after heart transplantation. Reactivation of Chagas disease can lead to graft failure or systemic complications such as fulminant central nervous system disease and sepsis. As such, careful screening for Chagas seropositivity prior to transplant is crucial to preventing negative outcomes in the post-transplant setting. One challenge in screening these patients is the variety of laboratory tests available and their differing sensitivities and specificities. In this case report, we present a patient who tested positive by a commercial *Trypanosoma cruzi* antibody assay and later tested negative by CDC confirmatory serological analysis. After the patient underwent orthotopic heart transplant, he underwent protocol-based polymerase chain reaction surveillance for reactivation as a result of persistent concerns for *T. cruzi* infection. It was discovered shortly thereafter that the patient had reactivation of Chagas disease, confirming that he did have Chagas cardiomyopathy prior to transplantation, despite negative confirmatory testing. This case illustrates the complexities of serological diagnosis of Chagas disease and the importance of additional testing for *T. cruzi* when the post-test probability remains high even with a commercial, negative serologic test.

## INTRODUCTION

Chagas cardiomyopathy carries a significant risk of reactivation that, when unrecognized, can cause lethal trypanosomiasis. Adequate screening is paramount to ensuring this risk is minimized. In patients with equivocal test results or patients with high pretest probability, we suggest that additional testing beyond the standard screening and confirmatory protocol is needed given the possibility of false-negative results. We present a case of post-transplant Chagas reactivation in a patient with a positive commercial *Trypanosoma cruzi* IgG ELISA, but negative confirmatory testing by the CDC.

## CASE PRESENTATION

A 50-year-old Hispanic gentleman from El Salvador with a history of nonischemic cardiomyopathy (ejection fraction, 20%), diabetes mellitus, hypertension, and anemia was transferred from an outside hospital after presenting with acute-on-chronic decompensated heart failure. He was evaluated by the advanced heart failure team for advanced therapies. During the hospital stay, he was treated with intravenous diuretics, but could not tolerate guideline-directed medical therapy as a result of hypotension. His laboratory workup revealed a brain natriuretic peptide level of 521 pg/mL; high-sensitivity troponin I, 43 ng/L; creatinine, 0.5 mg/dL; hemoglobin, 11 g/dL; and *T. cruzi* antibody IgG, 4.3 (IV normal, < 1). Electrocardiogram showed a sinus rhythm with a right bundle branch block and a left anterior fascicular block. The patient’s echocardiogram showed a left ventricular ejection fraction of < 20% with regional hypokinesis, moderately reduced right ventricular function, biatrial enlargement, severe tricuspid regurgitation, and mild mitral regurgitation. Chest X-ray showed pulmonary vascular congestion suggestive of pulmonary edema. He had a prior left heart catheterization that showed normal coronaries.

As an outpatient, the patient was monitored in the Advanced Heart Failure Clinic to complete the workup of his cardiomyopathy. Surprisingly, the *T. cruzi* confirmatory test from the CDC had a negative result (Weiner enzyme immunoassay, 0.003; negative trypomastigote excreted-secreted antigen blot). After discussing his case with the CDC, it was felt that repeat testing was warranted given his high post-test probability. However, at that time, the CDC parasitology laboratory was closed, delaying this process. He went on to experience further decline in his clinical status while on home inotropes resulting in multiple admissions for heart failure exacerbations, and he was eventually listed for heart transplant. He underwent successful orthotopic heart transplant (cytomegalovirus donor+/recipient+, Epstein-Barr virus donor+/recipient+) 3 months after his positive screening test, and his postoperative course was largely uncomplicated. Pathology examination of his explanted heart revealed chronic myocarditis with fibrosis and regenerative changes. For induction of immunosuppression, he was started on mycophenolate and high-dose steroids.

Because of persistent concerns for *T. cruzi* infection, and in collaboration with the CDC, a decision was made to perform protocol-based polymerase chain reaction (PCR) testing for reactivation of *T. cruzi* infection. Five weeks post-transplant, his PCR test showed a positive result, confirming *T. cruzi* infection, and a subsequent positive PCR test with a decrease in cycle threshold confirmed reactivation. During outpatient follow-up, the patient was recovering well and reporting minimal symptoms. Because his positive PCR result, he was referred to an infectious disease clinic, which recommended a 60-day course of benznidazole, 150 mg twice daily. He was placed on a protocol of weekly Chagas PCR testing until two sequential negative results returned and, after completion of his treatment, he had two separate negative PCR results. He experienced flushing with his first dose of benznidazole, but otherwise tolerated it well. Serial endomyocardial biopsies showed mild, acute cellular rejection (grade 1R), suggesting a favorable response to post-transplant immunosuppression and no evidence of significant graft rejection.

## DISCUSSION

Patients with Chagas cardiomyopathy are known to face a significant risk of reactivation after heart transplantation that, in turn, can cause significant detrimental consequences, including graft failure, increased morbidity, and increased mortality if not identified early during the course of post-transplantation.^1^ For this reason, appropriate diagnosis of Chagas cardiomyopathy prior to transplant is crucial to prevent negative outcomes in the post-transplant setting. At the time of publication, although there are four Food and Drug Administration–approved antibody detection assays for *T. cruzi* infection, commercial laboratories offer only two.^2^ Associated regional and university pathologists (ARUP laboratories) ARUP currently provides the Hemagen ELISA, and Quest and the Mayo Clinic provide Weiner v.3 ELISA. None of these laboratories offers confirmatory testing, and confirmation is chiefly through the CDC.^3^^,^^4^ Although test characteristics for these assays are published, there remain concerns about their accuracy for patients in whom *T. cruzi* I, rather than *T. cruzi* II, infection is suspected.^5^^,^^6^ Moreover, in patients with high pretest probability (based on endemic status, clinical presentation, and electrocardiographic and echocardiographic findings), the post-test probability may still be high enough to warrant additional testing. In this case, there was both a suspicion of laboratory error (i.e., testing the wrong patient sample), or concern that, in the setting of *T. cruzi* I infection, the CDC assays may have rendered a false-negative result.

Identifying patients with Chagas disease prior to heart transplantation is very important because it may alter the choice and dose of immunosuppression during the post-transplant period.^7^ There are no large studies comparing the various immunosuppression regimens in patients with Chagas cardiomyopathy; however, a greater number of reactivation cases have been diagnosed with the use of mycophenolate mofetil^7^^,^^8^ (which was used for the patient in this case). This raises the question of whether the post-transplant immunosuppression regimen should be altered or adjusted for patients found to have Chagas reactivation.

Another important aspect of proper identification of Chagas disease during the peritransplant setting is that it allows for the timely introduction of antiparasitic therapy and PCR monitoring for clearance of the disease. In many endemic Latin American countries, the approach to Chagas disease reactivation after solid organ transplant has been to wait for clinical manifestations before initiating treatment. There is evidence to suggest that clinical manifestations lag behind reactivation by days to weeks. As a result, in the United States, the standard is to treat with benznidazole immediately after making the diagnosis of Chagas disease reactivation and to screen serially for conversion to PCR negativity.^9^

Overall, patients with Chagas cardiomyopathy represent a population that benefits greatly from heart transplantation, with similar outcomes compared with non-Chagas cardiomyopathies. Chagas reactivation, however, is a major problem and, in the United States, reactivation has been found in 61% of transplant patients evaluated by the CDC.^10^

## CONCLUSION

Overall, because of the imperfection of current *T. cruzi* tests, repeat testing should be warranted for people with high-pretest probability despite a negative initial test result. Given that Chagas reactivation causes a major problem during the post-transplant period, to protect against reactivation, patients must be screened appropriately for Chagas disease and undergo serial testing during the post-transplant period, along with benznidazole therapy, if indicated, as the resultant myocarditis of Chagas reactivation can lead to graft failure. We present a patient from a Chagas-endemic area who tested positive initially for *T. cruzi*, but later tested negative on CDC confirmatory assays leading up to heart transplantation. Because of a high index of suspicion, he underwent serial PCR testing, leading to retrospective diagnosis of Chagas cardiomyopathy as well as acute reactivation. This unique case highlights the importance of a carefully selected screening process and retesting of patients with high pretest probability.
